# First gas chromatography-mass spectrometry (GC–MS) method for the detection and quantification of 11 trichothecenes and zearalenone in wheat plant-based beverages

**DOI:** 10.1016/j.fochx.2025.103449

**Published:** 2025-12-26

**Authors:** Ziyang Jia, Wenqi Huang, Kaifeng Zhao, Celia Costas, Maria Garcia-Marti, Jesus Simal-Gandara, Paz Otero

**Affiliations:** a Universidade de Vigo, Nutrition and Bromatology Group, Analytical Chemistry and Food Science Department, 36310 Vigo, Spain; b Instituto de Agroecoloxía e Alimentación (IAA), Universidade de Vigo, Campus Auga, 32004 Ourense, Spain; c CISPAC, Fontan Building, City of Culture, 15702 Santiago de Compostela, Spain; d Agricultural and Food Research Group (AA1), Galicia Sur Health Research Institute (IIS Galicia Sur). SERGAS-UVIGO, 36312 Vigo, Spain

**Keywords:** Trichothecenes, Zearalenone, QuEChERS, GC–MS, Wheat plant-based beverage

## Abstract

The presence of mycotoxins in plant-based beverages has not been regulated despite the potential presence of mycotoxins from the use of contaminated raw materials. This study reports the first gas chromatography–mass spectrometry (GC–MS) method developed for the rapid analysis of 12 common *Fusarium* mycotoxins in wheat plant-based beverages: fusarenon-X (FUS-X), nivalenol (NIV), diacetoxyscirpenol (DAS), HT-2, neosolaniol (NEO), T-2 triol, T-2 tetraol, deoxynivalenol (DON), 3-acetyldeoxynivalenol (3-ADON), 15-acetyldeoxynivalenol (15-ADON), T-2 and zearalenone (ZEA). The method is based on a QuEChERS extraction and, after optimization of chromatographic performance with two derivatization reagents, was validated in terms of linearity, matrix effect, limits of detection (LODs) and quantification (LOQs), exhibiting satisfactory recoveries above 72.49 % for all mycotoxins. Moreover, the multi-toxin method has many advantages such as easy operation, inexpensive cost and rapid analysis, constituting an effective choice for the monitoring of mycotoxins in wheat-based beverages whose consumption has increased significantly in recent years.

## Highlights

• A GC–MS method for rapid determination of mycotoxins was developed.
• The GC–MS was applied to commercial wheat plant-based beverages for the first time.
• The new GC–MS methodology was validated for 12 mycotoxins, including T-2 and HT-2.

## Graphical abstract

**Figure:**
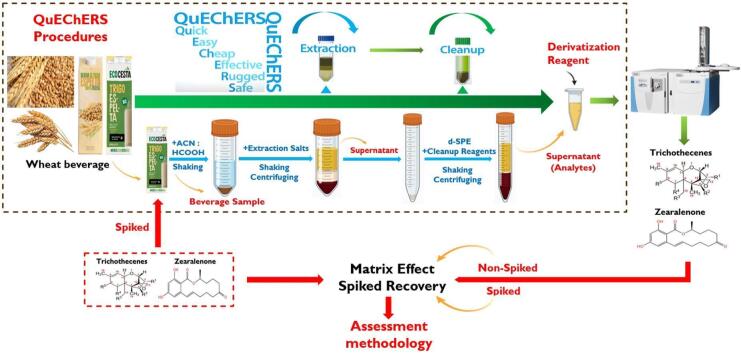

## 1 Introduction

Trichothecenes (TCTs) are a group of potentially toxic secondary metabolites produced by filamentous fungi such as *Fusarium, Trichthecium, Myrothecium, Stachybotrys* and *Xylaria* (Proctor et al., 2020). These toxins are primarily found in cereal grains, including wheat and corn, as well as their processed products. Exposure to TCT-contaminated crops can lead to a variety of metabolic, physiological, and immunological disorders in animals and humans, as well as acute and chronic diseases (Escrivá et al., 2015). Among the fungi that produce mycotoxins, *Fusarium* spp. are important plant pathogens that cause long-term epidemics of Fusarium head blight (FHB) on wheat and barley causing severe damage to crops, and serious consequences worldwide in terms of reduced crop yields and quality, and even more so in terms of threats to human and animal health (Goswami & Kistler, 2004; Huang et al., 2024). Common mycotoxins that cause FHB are deoxynivalenol (DON) and T-2 toxin (T-2). In parallel, contamination of animal feed with zearalenone (ZEA) is a global concern. Low doses of ZEA in feed can cause reproductive hormone disruption and organ damage in sows (Zhou et al., 2022).

Commonly, TCTs can be categorized into four types, A-, B-, C- and D-type, according to the type and number of functional groups, with A-type and B-type being the most common. A-type TCTs have hydroxyl, ester or unsubstituted groups at the C-8 site around the core ring (Desjardins, 2009). Typical representatives include neosolaniol (NEO), diacetoxyscirpenol (DAS), T-2, T-2 triol and HT-2 toxin (HT-2). It is worth mentioning that T-2 is the most toxic of the A-type TCTs known to date (Yang et al., 2020). In addition, B-type TCTs is categorized by the presence of a carbonyl-substituted functional group at the C-8 position around the core ring, and typical representatives including DON, nivalenol (NIV), 3-acetyldeoxynivalenol (3-ADON), 15-acetyldeoxynivalenol (15-ADON), fusarenon-X (FUS-X) and others (Suzuki & Iwahashi, 2012). Of these, DON and its acetylated forms are among the most widespread contaminants in cereals and have attracted particular attention due to their frequent occurrence (Ran et al., 2013). Despite TCTs, the mycotoxin ZEA can be also present in wheat and wheat-derived products. ZEA is mainly also produced by *Fusarium* spp. such as *F. graminearum (Gibberella zeae), F. culmorum, F. crookwellense, F. semitectum and F. equiseti* species of fungi (Rai, Mukul and Tripathi, 2020).

**Fig. 1:**
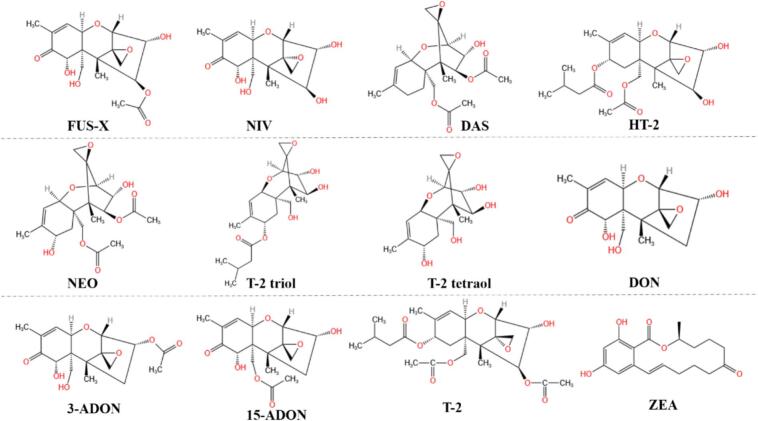
Molecular structural formulae of the 12 mycotoxins: fusarenon-X (FUS-X), nivalenol (NIV), diacetoxyscirpenol (DAS), HT-2 toxin (HT-2), neosolaniol (NEO), T-2 triol, T-2 tetraol, deoxynivalenol (DON), 3-acetyldeoxynivalenol (3-ADON), 15-acetyldeoxynivalenol (15-ADON), T-2 toxin (T-2) and zearalenone (ZEA).

Due to their adverse health effects, both TCTs and ZEA must be carefully monitored to minimize health risks to consumer health and to maintain productivity. Fig. 1 illustrates the molecular structures of main TCTs and ZEA. Due to the widespread public health and agricultural impact of mycotoxin contamination caused by the fungus *Fusarium* spp., the EU has established limits to control contamination levels. Currently, the Commission Regulation (EU) 2023/915 sets the maximum limits of mycotoxins in food products (none are wheat plant-based beverages). It established 750 and 75 μg/kg for DON and ZEA, respectively, in cereals (other than rice, milling products of maize and baby food placed on the market for the final consumer (EC 2023/915, 2023). Moreover, the Commission Regulation (EU) 2024/1022 reduced the limits of DON with respect to the Regulation UE 2023/915 in some food matrices (EC 2024/1022, 2024). For instance, in bakery products, the DON limit decreased from 500 to 400 μg/kg, in pasta products from 650 to 600 μg/kg and in infant products from 200 to 150 μg/kg. Besides, the new Commission Regulation (UE) 2024/1038 includes the limits for two new toxins (T-2 y HT-2) in several foods (EC 2024/1038, 2024), including cereals like wheat and cereal-based foods like bakery products, breakfast cereals and pasta products being the limit 20 μg (T-2 + HT-2)/kg. However, none of the foods are wheat plant-based beverages.

Efforts to detect mycotoxins in wheat have been ongoing for a long time. These efforts are aimed at ensuring the safety and quality of wheat and its products, particularly for the possible presence of major mycotoxins such as Fusarium mycotoxins. Gas chromatographic (GC) separation techniques have been used for the detection and analysis of TCTs analogs as early as the 1980s (Stahr et al., 1985). In recent decades, more efficient analytical methods (higher detection sensitivity and resolution) have been progressively established, making the GC method a solid option for the qualitative and quantitative determination of TCTs, besides the LC-MS/MS methodology. In addition, the GC–MS method has been widely used for the detection of mycotoxins contamination in other cereals and their derived foods, showing good separation performance and trace analytical capability, such as milled grain-based products including rice, maize, spelt, oat, soy and tapioca (Rodríguez-Carrasco et al., 2014).

In this context, the objective of this paper is to develop a new GC–MS for the detection of mycotoxins in wheat plant-based beverages. The consumption of plant-based beverages has surged in recent years as part of a shift toward alternative and health-conscious lifestyle (Lawrence et al., 2016). The rapid development of food science and technology in the current society was accompanied by the expansion of the functional food market, where more nutritious, beneficial and innovative food products are gradually entering the market to satisfy consumers' needs. The global size of plant-based beverages increased at a growth rate of 4.91 % per annum to reach more than USD 12.1 billion by 2024 (Penha et al., 2021). However, these new products can have mycotoxins, especially if they are present in the raw material as is the case of wheat plant-based beverages. Thus, new methodology for the analysis of mycotoxins in wheat plant-based beverages is urgently needed. So far, the few available methods to detect mycotoxins in this kind of matrix are based on LC-MS/MS (Kerstner & Garda-Buffon, 2024; Miró-Abella, Herrero, Canela, Arola, Borrull, Ras, et al., 2017). This technique against GC–MS has some disadvantages, such as weaker separation ability, worse selectivity, higher sample usage, and slower analysis. Moreover, these methods are mainly validated for matrices such as oat, rice, coconut, almond, birdseed, soybean, and soy–rice blends (Arroyo-Manzanares et al., 2019; Hamed et al., 2017b; Hamed et al., 2019; Miró-Abella et al., 2017). Therefore, this study aimed to optimize and validate a new method to analyze mycotoxins in wheat beverages and to research the presence of mycotoxins in commercial wheat plant-based beverages.

## 2 Materials and methods

### 2.1 Chemicals and reagents

The following analytical mycotoxins standards were used in the study: FUS-X, NIV, DAS, HT-2, NEO, T-2 triol, T-2 tetraol were provide by Dr. Ehrenstorfer (Wesel, Germany), which were both at 100 μg/mL in ACN except T-2 triol and T-2 tetraol (both at 50 μg/mL in ACN). In addition, the standards of DON, 3-ADON, 15-ADON, T-2 and ZEA were obtained from Romer Labs (Runcorn, UK). Individual stock solutions of TCTs and ZEA were prepared at the same concentration (100 μg/mL) in ACN (T-2 triol and T-2 tetraol were 50 μg/mL in ACN), while the work solutions of all mycotoxins were prepared in 10 μg/mL in ACN. All standards were stored in darkness and kept at −20 °C until the GC–MS analysis.

Water was purified in a Milli-Q system (Merck Millipore S.A.S, Molsheim, France). The chemicals used in the study were purchased from the following suppliers: the n-hexane and formic acid were purchased from Merck KGaA (Darmstadt, Germany). The solvents acetonitrile (ACN) and acetone were purchased from CARLO ERBA (Barcelona, Spain). Anhydrous magnesium sulfate (MgSO_4_), sodium chloride (NaCl), Na-Citrate, disodium citrate sesquihydrate, primary secondary amine (PSA) and octadecyl silica-bonded sorbent (C18) were purchased from Thermo Fisher Scientific (Waltham, Massachusetts, USA). The derivatization reagent (Tri-Sil TBT) composed of BSA (N,O-bis (trimethylsilyl)acetamide) + TMCS (trimethylchlorosilane) + TMSI (*N*-trimethylsilyimidazole) (3,2,3) was purchased from Thermo Fisher Scientific (Waltham, Massachusetts, USA). N,O-bis (trimethylsilyl)acetamide was obtained by Thermo Fisher Scientific (Waltham, Massachusetts, USA).

Additionally, the wheat plant-based beverage was purchased from the local market in Vigo, Spain. The product contains 16 % *Triticum spelta*, which is nutritionally superior to regular wheat and is still grown in Central Europe. This is a commercially available organic spelt-wheat plant-based beverage produced in Madrid, composed of water, 16 % spelt, cold-pressed sunflower oil, and salt. The nutritional information per 100 g is 1.1 g fat, 10.5 g carbohydrates, 5.5 g sugars, 0.8 g protein, 0.9 g fiber, and 0.08 g salt (total 57 kcal). During method development, several batches of this beverage were tested, but all with the same composition. In fact, the composition of wheat-based plant beverages, commercialized under different brands, is also similar. For example, the main characteristic compound of this product, the spelt wheat, usually ranges from 15 to 17 % depending on the brand.

### 2.2 Optimization of derivatization conditions

Sample derivatization conditions were optimized as follows. First, two commonly used derivatization reagents, Tri-Sil TBT and BSA, were tested under identical conditions (Method 1). Briefly, the mixture of 12 mycotoxins at 2 μg/mL was evaporated with a centrifugal vacuum concentrator (VC2124, Gyrozen Co., Ltd., Korea) at 37 °C, 1900 rpm for 2 h. And 30 μL of Tri-Sil TBT or BSA was added and kept at 80 °C for 20 min. After that, the derivatized sample was diluted with 270 μL of n-hexane. Moreover, the sample was transferred to an autosampler vial for the GC–MS analysis. Next, the effects of derivatization temperature and time were assessed under two different sets of conditions: the condition used in Method 1 (80 °C for 20 min) and a milder condition (Method 2). Method 2 used the same conditions as Method 1, only changing the reaction time and temperature to 25 °C for 30 min.

### 2.3 Sample preparation

Sample preparation was performed according to a previous study based on the QuEChERS method, with some modifications (Juan et al., 2017; Miró-Abella et al., 2017). Briefly, 10 mL of wheat plant-based beverage was homogenized and transferred into the tube containing 10 mL of ACN with 0.5 % formic acid. The tube was shaken vigorously and vortexed to ensure adequate mixing. Subsequently, the extraction salts including 4 g anhydrous MgSO_4_, 1 g NaCl, 1 g Na-Citrate and 0.5 g disodium citrate sesquihydrate, were added to the tube. The mixture was shaken vigorously and centrifuged at 5000 rpm for 5 min (MEYA-16, Remi Elektrotechnik Ltd., Vasai, India). The supernatant was submitted to a dispersive solid phase extraction (d-SPE) with a tube containing 900 mg MgSO_4_, 150 mg PSA and 150 mg C18. Likewise, was shaken vigorously and centrifuged at 5000 rpm for 5 min. The supernatant was centrifuged at 14000 rpm for 5 min and collected for derivatization. 300 μL of the final supernatant was evaporated with a centrifugal vacuum concentrator (VC2124, Gyrozen Co., Ltd., Korea) at 37 °C, 1900 rpm for 2 h. Sample was derivatized using Method 2 with Tri-Sil TBT. After that, the derivatized sample was diluted with 270 μL of n-hexane. Moreover, the sample was transferred to an autosampler vial for the GC–MS analysis.

### 2.4 GC–MS conditions

A Thermo Fisher Scientific TRACE 1300 GC system, coupled with a Thermo Fisher Scientific ISQ 7000 single quadrupole mass spectrometer equipped with an inert electron-impact ion source, and a Thermo Fisher Scientific AI/AS 1310 autosampler (Thermo Fisher Scientific, Massachusetts, USA) was utilized for mass spectrometry (MS) analysis. The temperatures of the transfer line and ion source were maintained at 280 °C and 230 °C, respectively. The mass spectrometer functioned using electron impact ionization (EI) at 70 eV. Nitrogen served as the collision gas for MS experiments, while helium, with a purity of 99.999 %, was employed as the quenching gas. Data acquisition and analysis were conducted using Thermo Fisher Scientific Chromatography 7 software. A Rxi-5 ms capillary column (30 m × 0.25 mm × 0.25 μm) was employed for the separation of compounds. One microliter of the final reconstituted mycotoxin extract solution was injected in splitless mode at 300 °C using a programmable temperature vaporization (PTV) inlet, with helium as the carrier gas at a constant pressure of 20.3 psi.

### 2.5 Optimization of toxin separation performance

**Fig. 2:**
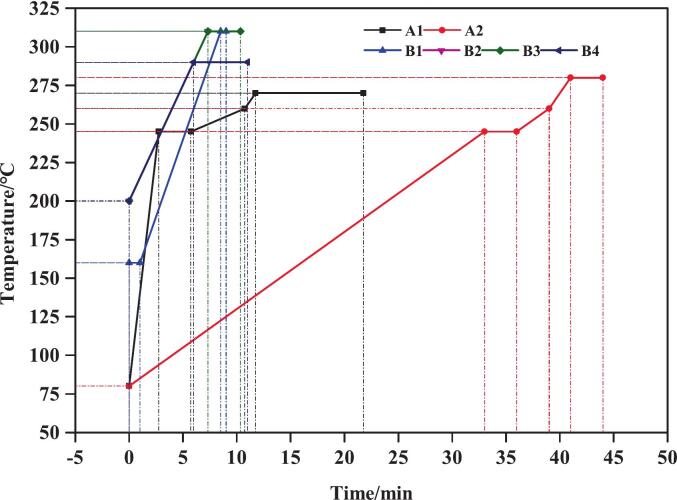
A1, A2, B1, B2, B3 and B4 represent six different column temperature programs. A1 and A2 include several temperatures climbing, while B1–4 only have once of it. A1: initial temperature at 80 °C, increased to 245 °C at 60 °C per min and hold for 3 min. Then, temperature was increased to 260 °C at 3 °C per min and immediately increased to 270 °C in 1 min, as well as hold for 10 min. A2: initial temperature at 80 °C, increased to 245 °C at 5 °C per min and hold for 3 min. Then, the temperature was increased to 260 °C at 5 °C per min and immediately increased to 280 °C in 2 min, as well as hold for 3 min. B1: initial temperature at 160 °C and hold for 1 min. Then, the temperature was increased to 310 °C at 20 °C per min and hold for 0.5 min. B2: initial temperature at 200 °C and was immediate increased to 310 °C at 15 °C per min. B3: initial temperature at 200 °C and was immediate increased to 310 °C at 15 °C per min, and kept at 310 °C for 3 min. B4: initial temperature at 200 °C and was immediate increased to 290 °C at 15 °C per min, and kept at 290 °C for 5 min.

To optimize the separation of mycotoxins, six different GC column temperature programs were evaluated, as illustrated in Fig. 2. These programs fall into two categories: (1) procedural temperature ramping involving multiple heating stages with varying rates (Programs A1 and A2), and (2) single-rate temperature ramps with different initial and final temperatures and total run times (Programs B1-B4). Program A1 began at 80 °C, increased to 245 °C at 60 °C/min and held for 3 min, followed by a rise to 260 °C at 3 °C/min and a rapid increase to 270 °C within 1 min, with a final hold of 10 min. Program A2 followed a similar pattern but with slower rates: starting at 80 °C, increasing to 245 °C at 5 °C/min and held for 3 min, then to 260 °C at 5 °C/min, followed by a 2 min ramp to 280 °C, and a final hold of 3 min. In contrast, the B-series programs adopted a simpler approach. Program B1 started at 160 °C (1 min hold), ramped to 310 °C at 20 °C/min, and held for 0.5 min. B2 started at 200 °C and ramped directly to 310 °C at 15 °C/min without a hold.

### 2.6 Method validation

The GC–MS method developed in this study was optimized and validated for the determination of 11 trichothecenes (TCTs) and zearalenone (ZEA) in wheat-based plant beverages. The method was evaluated in terms of linearity, limits of detection (LODs), limits of quantification (LOQs), matrix effect, accuracy and precision.

Linearity was assessed by calculating the correlation coefficients (R^2^) of different calibration curves of mycotoxins constructed in both solvent (acetonitrile) and blank matrix. Six to nine concentration levels were set for each mycotoxin, covering the expected contamination levels in plant-based beverages.

LODs and LOQs were determined based on the standard deviation of blank matrix extracts and the slope of the calibration curves, in accordance with the procedure described by Ferreira et al.(Ferreira et al., 2012). These parameters were calculated based on the standard deviation (σ) of the response from blank samples (*n* ≥ 10) and the slope (S) of the calibration curve, according to the following equations: LOD = 3.3 × (σ/S) and LOQ = 10 × (σ/S). This approach provides a statistically sound estimation of method sensitivity by accounting for both instrumental variability and analytical responsiveness.

To further evaluate the matrix effect, signal suppression/enhancement (SSE) was measured in wheat-based plant beverage extracts. To investigate the signal suppression/enhancement (SSE) caused by the matrix, standard solutions and matrix-matched calibration were used to study the matrix effect. In such a way, calibration curves at least 6 concentration levels using pure solvent or non-toxic extract matrix were constructed. The SSE was calculated using the slopes of the curves, which according to the following equation: *SSE (%) = slope of spiked extracts curve/slope of standards curve in solution×100 %.* SSE value was equal to 100 %, no matrix effect was observed, whereas a value higher than 100 % meant a positive matrix effect due to an enhancement of the ionization. If this value was less than 100 %, there was a negative effect, which entailed a suppression of the signal due to ionic suppression.

The accuracy and precision of the method were evaluated through recovery experiments by spiking blank samples with mycotoxins at two concentration levels (250 ng/mL and 500 ng/mL). Briefly, the amount of 10 mL of wheat plant-based beverage was spiked with FUS-X, NIV, DAS, HT-2, NEO, T-2 triol, T-2 tetraol, DON, 3-ADON, 15-ADON, T-2 and ZEA with the amount of 2500 ng or 5000 ng, then followed by QuEChERS extraction and analyzed by GC–MS.

Recovery experiments were performed in triplicate on the same day to assess repeatability. The recovery and the relative standard deviation (RSD) were calculated to evaluate precision. Finally, the accuracy was assessed by determining the recovery of the extraction.

## 3 Results and discussion

The occurrence of mycotoxins in plant-based beverages has not been legislated, consequently, there is not much research about the presence of mycotoxins in these products. The few commercial matrices tested were oat, rice, coconut, almond, birdseed, soybean, soy and rice beverages in which mycotoxins were found in some of them. For example, DON was observed in oat beverages at concentrations of 191–270 μg/L (Hamed et al., 2017a). The data reveals a matter of concern, indicating that proper control should be applied to these functional products, whose consumption has recently increased (Lawrence et al., 2016). Thus, this study aimed to develop a GC–MS method applicable to the analysis of wheat-based beverages and, to carry out a preliminary study on the incidence of toxins in these products.

### 3.1 Optimization of derivatization conditions

Prior to the analysis of mycotoxins in wheat plant-based beverage products, the GC–MS method was optimized by evaluating the sample derivatization conditions and the GC column temperature programs.

**Fig. 3:**
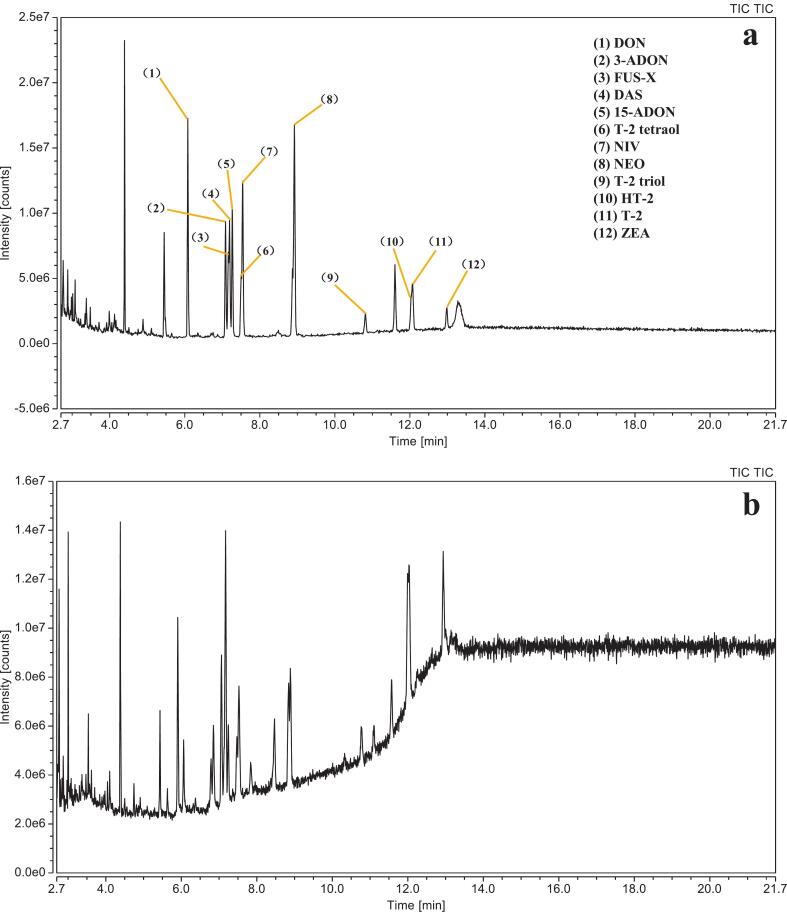
Gas chromatogram (GC–MS) analysis in total ion chromatogram (TIC) mode of the 12 mycotoxins at 4 μg/mL. (a) The mycotoxins derivatized by Tri-Sil TBT; (b) The mycotoxins derivatized by BSA. The chromatographic column was an Restek Rxi-5 ms capillary column (30 m × 0.25 mm × 0.25 μm). Oven program: initial temperature at 80 °C, increased to 245 °C at 60 °C per min and hold for 3 min. Then, temperature was increased to 260 °C at 3 °C per min and immediate increased to 270 °C in 1 min, as well as hold for 10 min (Run time = 21.75 min). One microliter of the final reconstituted mycotoxin extract solution was injected in splitless mode at 300 °C using a programmable temperature vaporization (PTV) inlet, with helium as the carrier gas at a constant pressure of 20.3 psi. The temperatures of the transfer line and ion source were 280 °C and 230 °C.

Although several methods of derivatization procedures have been reported for mycotoxin analysis, a direct comparison of their efficiency is lacking (Ferreira et al., 2012; Rodríguez-Carrasco et al., 2014). Therefore, we initially compared the performance of two commonly used derivatization reagents, Tri-Sil TBT and BSA. For this, toxins were derivatized with both reagents following identical protocol (showed in material and methods, Method 1) and then, the signal intensity of the mycotoxins were compared. The chromatograms of the derivatized mycotoxins are shown in Fig. 3. Under identical derivatization temperature and time conditions, Tri-Sil TBT yielded significantly higher signal intensities than BSA. Moreover, the chromatogram obtained using BSA displayed poor peak resolution compared to those obtained using Tri-Sil TBT. Tri-Sil TBT, which contains TMCS and TMSI as catalytic agents, provided higher derivatization efficiency than BSA, likely due to its enhanced reactivity in silylation reactions. Therefore, Tri-Sil TBT was selected for subsequent method development.

**Fig. 4:**
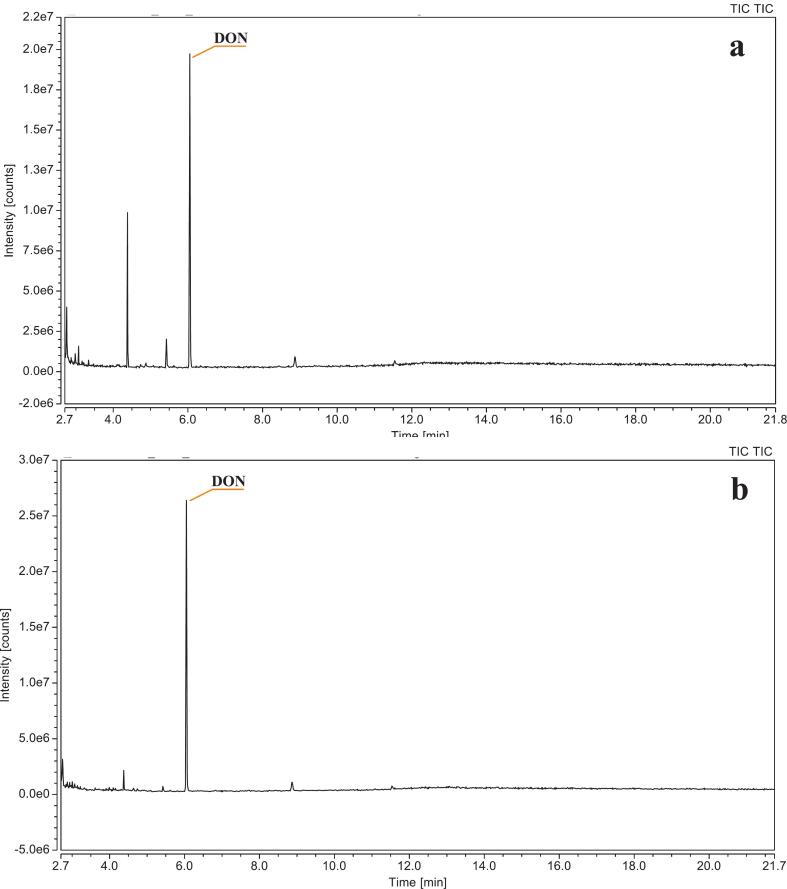
Gas chromatogram (GC–MS) analysis in total ion chromatogram (TIC) mode of DON at two different derivatization temperature. (a) The DON derivatized at 80 °C for 20 min; (b) The DON derivatized at 25 °C for 30 min. The chromatographic column was an Restek Rxi-5 ms capillary column (30 m × 0.25 mm × 0.25 μm). Oven program: initial temperature at 80 °C, increased to 245 °C at 60 °C per min and hold for 3 min. Then, temperature was increased to 260 °C at 3 °C per min and immediate increased to 270 °C in 1 min, as well as hold for 10 min (Run time = 21.75 min). One microliter of the final reconstituted mycotoxin extract solution was injected in splitless mode at 300 °C using a programmable temperature vaporization (PTV) inlet, with helium as the carrier gas at a constant pressure of 20.3 psi. The temperatures of the transfer line and ion source were 280 °C and 230 °C.

Next, the effects of derivatization temperature and time were assessed under two different sets of conditions: Method 1 and Method 2 (described in material and method section), since both temperature and duration have a notable impact on reaction efficiency (Bowden et al., 2009). These parameters were selected based on previous studies (Ferreira et al., 2012; Rodríguez-Carrasco et al., 2013). The intensity of derivatized mycotoxins was also evaluated. As shown in Fig. 4, the intensity of DON derivatized at 25 °C for 30 min (2.6e^7^) was notably higher than that at 80 °C for 20 min (2.0e^7^), indicating that lower temperature with longer reaction time led to improved derivatization efficiency.

### 3.2 Optimization of toxin separation performance

To optimize the separation of mycotoxins, six different GC column temperature programs were evaluated, as illustrated in Fig. 2 and explained in material and method section. Among all tested conditions, Program A1 provided the best separation performance and it was subsequently used as a basis for optimizing the final method of 15.75 min runtime. Fig. 2 depicts the original Program A1, which had a total runtime of 21.75 min, including a 10 min hold at 270 °C. Subsequent experiments indicated that all target mycotoxins eluted within 13 min. Therefore, the hold-time at 270 °C was shortened, resulting in the optimized Program A1 with a total runtime of 15.75 min, as used in all reported experiments. This optimization reduced the total runtime, improving analytical efficiency, and without modifying the separation performance of target mycotoxins. In addition, the retention time (R_t_) and characteristic ions of mycotoxins for GC–MS analysis were detailed in Table 1.

**Table 1:** Gas chromatographic (GC–MS) analysis parameters for the mycotoxins analysis. Fusarenon-X (FUS-X), nivalenol (NIV), diacetoxyscirpenol (DAS), HT-2 toxin (HT-2), neosolaniol (NEO), T-2 triol, T-2 tetraol, deoxynivalenol (DON), 3-acetyldeoxynivalenol (3-ADON), 15-acetyldeoxynivalenol (15-ADON), T-2 toxin (T-2), zearalenone (ZEA), Retention time (Rt).

Mycotoxins	Rt/min	Characteristic ions/*m/z*
FUS-X	6.93	245, 260, 450, 480, 555
NIV	7.29	191, 289, 379
DAS	6.98	124, 289, 350, 379
HT-2	11.68	157, 185, 347, 466
NEO	8.56	167, 195, 252
T-2 triol	10.42	259, 287, 598.5
T-2 tetraol	7.25	231, 259, 349, 393
DON	5.92	235, 259, 295, 392, 407, 422, 512
3-ADON	6.87	287, 295, 377, 392, 467
15-ADON	7.05	235, 295, 350, 392
T-2	11.72	122, 244, 259, 350
ZEA	12.56	151, 333, 429, 462

**Fig. 5:**
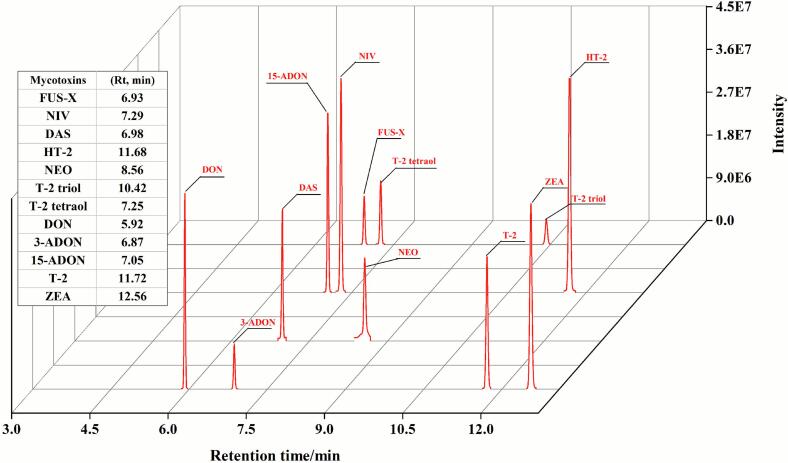
Gas Chromatography (GC–MS) analysis in selected ion monitoring (SIM) mode of the 12 mycotoxins at 4 μg/mL, which are fusarenon-X (FUS-X), nivalenol (NIV), diacetoxyscirpenol (DAS), HT-2 toxin (HT-2), neosolaniol (NEO), T-2 triol, T-2 tetraol, deoxynivalenol (DON), 3-acetyldeoxynivalenol (3-ADON), 15-acetyldeoxynivalenol (15-ADON), T-2 toxin (T-2) and zearalenone (ZEA). Standard was dissolved in acetonitrile. The chromatographic column was an Restek Rxi-5 ms capillary column (30 m × 0.25 mm × 0.25 μm). Oven program: initial temperature at 80 °C, increased to 245 °C at 60 °C per min and hold for 3 min. Then, temperature was increased to 260 °C at 3 °C per min and immediate increased to 270 °C in 1 min, as well as hold for 4 min (Run time = 15.75 min). One microliter of the final reconstituted mycotoxin extract solution was injected in splitless mode at 300 °C using a programmable temperature vaporization (PTV) inlet, with helium as the carrier gas at a constant pressure of 20.3 psi. The temperatures of the transfer line and ion source were 280 °C and 230 °C.

Representative GC–MS chromatograms of the 12 mycotoxins at 4 μg/mL are presented in Fig. 5. As it can be observed, the 12 mycotoxins were properly identified and separated using a new GC method, which employed a Rxi-5 ms capillary column (30 m × 0.25 mm × 0.25 μm) under a gradient temperature program ranging from 80 °C to 270 °C, following derivatization with Tri-Sil-TBT at 25 °C for 30 min. From our knowledge, this is the first study utilizing an Rxi-5 ms capillary column for the detection of mycotoxins. Previous GC–MS methods typically employed HP-5MS capillary column for mycotoxin determination in cereal matrices (Rodríguez-Carrasco et al., 2012; Rodríguez-Carrasco et al., 2014). This can be an operation advance considered for many laboratories which uses this column for the detection of other contaminants or compounds as is the case of isobutane oxidation products, marijuana degradation products or haloanilines (Di Nunzio et al., 2024; Willms et al., 2016; Zhang et al., 2022) and now they can expand to mycotoxins. The **change and installation of GC column** is a challenge and takes time. So, this column can be used to detect mycotoxins in many labs that are already using it for other molecules.

### 3.3 Method validation

The linearity of the method was evaluated by preparing mycotoxin standards at different concentrations in the ACN. Calibration curves were constructed using at least six concentration levels for each mycotoxin, and the coefficient of determination (R^2^) was calculated based on a linear regression model. As shown in Table 2, all mycotoxins exhibited excellent linearity, with R^2^ values exceeding 0.999.

**Table 2:** Performance characteristics of the analysis method in solvent. Fusarenon-X (FUS-X), nivalenol (NIV), diacetoxyscirpenol (DAS), HT-2 toxin (HT-2), neosolaniol (NEO), T-2 triol, T-2 tetraol, deoxynivalenol (DON), 3-acetyldeoxynivalenol (3-ADON), 15-acetyldeoxynivalenol (15-ADON), T-2 toxin (T-2), zearalenone (ZEA), limit of detection (LOD), limit of quantification (LOQ), coefficient of determination (R^2^).

Mycotoxins	LOD/ ng/mL	LOQ/ ng/mL	Calibration range/ ng/ml	Calibration curves	R^2^
FUS-X	28.77	87.17	62.5–3000	y = 94,974× + 3518.5	0.9998
NIV	5.31	16.11	125–3000	y = 422,740× + 31,484	0.9995
DAS	11.84	35.89	62.5–2000	y = 238,013× + 3192.9	0.9999
HT-2	7.73	23.44	62.5–2000	y = 6,600,112× + 8109.5	0.9999
NEO	14.00	42.42	125–4000	y = 179,410×-2963.9	0.9998
T-2 triol	28.52	86.44	125–3000	y = 69,697× + 3300.6	0.9994
T-2 tetraol	9.51	28.82	125–3000	y = 132,843× + 21,398	0.9998
DON	9.44	28.61	62.5–2000	y = 245,088×-2106.3	0.9993
3-ADON	14.23	43.11	62.5–2000	y = 69,120× + 4158.3	0.9995
15-ADON	4.38	13.27	62.5–2000	y = 337,902× + 5021.8	0.9999
T-2	11.96	36.25	62.5–2000	y = 293,506×-6380.7	0.9996
ZEA	9.37	28.40	62.5–2000	y = 422,441× + 8028.1	0.9992

The sensitivity of the developed method was evaluated through the determination of LOD and LOQ. For the 12 mycotoxins analyzed, the calculated LODs ranged from 4.38 to 28.77 ng/mL, while LOQs spanned 13.27 to 87.17 ng/mL (Table 2). These results indicate that the method exhibits sufficient sensitivity for detecting and quantifying mycotoxins at trace levels. Variations in LOD and LOQ among the analytes may result from differences in their physicochemical properties, ionization efficiencies, and potential matrix interactions. Such sensitivity ensures reliable detection even at trace levels, critical for monitoring mycotoxin contamination in complex food matrices. The method's sensitivity surpasses previously reported techniques for simultaneous mycotoxin analysis, underscoring its advancement in multi-analyte detection. For further validation, future studies may consider the use of certified reference materials to enhance metrological traceability and ensure regulatory compliance.

In order to evaluate the GC method in a food matrix, wheat plant-based beverage samples were selected, as mycotoxins in such products may pose potential health risks to consumers. Initially, the beverage was directly analyzed to assess the natural presence of mycotoxins. The results showed that no mycotoxins were detected in the commercial beverage, allowing them to be considered as blank matrices for further validation. Subsequently, blank beverage was spiked with the 12 mycotoxins. Matrix effects represent a critical factor in GC–MS quantitative analysis, as they can lead to ion suppression or enhancement, thereby affecting the accuracy and reproducibility of the results. Furthermore, establishing a compatible and universal sample pretreatment protocol is essential for achieving reliable detection. Recently, the QuEChERS extraction procedure has been recognized as an effective and classical method for the extraction of mycotoxins from cereal matrices, showing high generality and good recovery results (Cunha & Fernandes, 2010; Ferreira et al., 2012). In the present study the QuEChERS method was used with the aim of providing a robust GC method to detect mycotoxins in wheat plant-based beverage, with an extraction method with high percentage of toxin recovery and without interferences of matrix. To quantify the matrix effects, the signal suppression/enhancement (SSE) values were calculated for each analyte. As illustrated in Table 3, SSE values exhibited significant analyte-dependent variation (90.1–129.4 %), indicating analyte-dependent variation and revealing three distinct response patterns. First, isolated suppression effect: NEO demonstrated moderate signal suppression (90.1 %), potentially attributable to disruptions from co-eluting chlorophyll derivatives or phenolic compounds commonly present in plant matrices. Second, neutral zone: FUS-X (106.8 %), DAS (105.3 %), T-2 triol (107.3 %), and T-2 tetraol (107.7 %) showed minimal matrix interference (SSE close to 100 %), suggesting effective compensation of ionization competition through optimized chromatographic separation at their specific retention windows. Third, pronounced enhancement cluster: 7 toxins displayed significant signal amplification, with NIV (110.0 %), HT-2 (112.7 %), DON (120.6 %), 3-ADON (123.6 %), 15-ADON (112.9 %), T-2 (119.6 %), and ZEA (129.4 %) showing exceptionally high enhancement. This phenomenon may stem from matrix derivatives-induced synergistic effects of beverage components stabilizing gas-phase ions. This comprehensive assessment of matrix effects provides essential validation for the applicability of the developed method in complex plant-derived beverages, ensuring accurate quantification across a broad range of mycotoxins.

**Table 3:** Performance of the analysis and extraction methods in wheat plant-based beverage. Fusarenon-X (FUS-X), nivalenol (NIV), diacetoxyscirpenol (DAS), HT-2 toxin (HT-2), neosolaniol (NEO), T-2 triol, T-2 tetraol, deoxynivalenol (DON), 3-acetyldeoxynivalenol (3-ADON), 15-acetyldeoxynivalenol (15-ADON), T-2 toxin (T-2), zearalenone (ZEA). Recovery (R) at two concentration levels, 0.25 μg/mL (R_1_) and 0.5 μg/mL (R_2_). Relative standard deviation (RSD), signal suppression/enhancement (SSE).

Mycotoxins	R_1_ ± RSD/%	R_2_ ± RSD/%	SSE/%
FUS-X	98.21 ± 11.77	92.75 ± 2.51	106.8
NIV	106.46 ± 6.82	102.12 ± 0.73	110.0
DAS	86.38 ± 2.62	105.83 ± 9.03	105.3
HT-2	97.18 ± 1.98	95.52 ± 5.64	112.7
NEO	111.73 ± 8.27	99.00 ± 6.39	90.1
T-2 triol	89.26 ± 8.60	111.60 ± 9.36	107.3
T-2 tetraol	91.55 ± 5.36	114.57 ± 13.81	107.7
DON	96.10 ± 1.86	81.62 ± 5.83	120.6
3-ADON	73.67 ± 2.47	82.23 ± 0.74	123.6
15-ADON	83.68 ± 8.10	89.83 ± 2.18	112.9
T-2	103.38 ± 0.81	92.81 ± 2.17	119.6
ZEA	72.49 ± 2.17	75.48 ± 3.50	129.4

The developed method demonstrated high recovery rates (Table 3**)** and satisfactory repeatability (Table 3**)** for the simultaneous determination of 12 mycotoxins in wheat plant-based beverages at two spiking levels: R_1_ (0.25 μg/mL) and R_2_ (0.5 μg/mL). Quantification was determined using calibration curves established with solvent-based standards. Accuracy was studied through recovery. Overall recoveries ranged from 72.49 % to 114.57 % across both spiked levels. Notably, 10 out of 12 mycotoxins exhibited recoveries within the range of 80–115 % for multi-analyte methods considered acceptable for multi-mycotoxin analysis (Liu et al., 2016; Mahmoud et al., 2018). In addition, precision was studied through RSD. At the level R_1_, recoveries showed RSD values ranging from 0.81 % to 11.77 %, while at the level R_2_, RSD values spanned 0.73 % to 13.81 %. Particularly satisfactory performance was observed for HT-2 (97.18 and 95.52 %), and T-2 (103.38 % and 92.81 %), demonstrating both high accuracy (90–105 %) and repeatability (RSD <6 %) in both R_1_ and R_2_ levels. While some compounds like NEO (111.73 ± 8.27 %) at the R_1_ level and T-2 triol (111.60 ± 9.36 %), T-2 tetraol (114.57 ± 13.81 %) at the R_2_ level showed marginally elevated recovery values, their repeatability remained within acceptable limits for complex multi-analyte methods. These variations may reflect differential matrix effects or extraction efficiencies among structurally diverse mycotoxin classes.

To date, there are few studies on the true content of various mycotoxins in wheat beverages. Most have focused on natural exposure studies of mycotoxins in plant-based beverages such as oat, rice, soy, and nuts. The method was tested in 10 mL wheat spiked with 2.5 or 5.0 μg of mycotoxins, yielding final concentrations of 0.25 or 0.5 μg/mL. To detect them, it was possible to design an adequate clean toxin recovery able to quantify the mycotoxins in the following ranges: 62.5 μg/L-2 mg/L for DAS, HT-2, DON, 3-ADON, 15-ADON, T-2, and ZEA; 125 μg/L-3 mg/L for NIV, T-2 triol, and T-2 tetraol; 125 μg/L-4 mg/L for NEO; and 62.5 μg/L-3 mg/L for FUS-X. The overall method performance meets validation criteria for multi-residue analysis, demonstrating sufficient reliability for quantitative determination of these mycotoxins in the tested concentration range. The slightly higher variation observed for certain mycotoxins at lower concentrations may be attributed to matrix effects or minor losses during sample preparation. Nevertheless, the method demonstrated satisfactory performance for mycotoxin quantification in the tested conditions.

Several LC-MS/MS methods for trichothecenes and zearalenone in plant-based beverages have been reported, including those of Kerstner and Garda Buffon (2024) and Miró-Abella et al. (2017), which demonstrated excellent sensitivity and robust quantitative performance. In the study by Miró-Abella et al. (2017), similar recoveries for DON, HT-2, T-2, and ZEA were reported across oat, soy, and rice beverage matrices. Specifically, DON recoveries were 87 %, 84 %, and 87 %; HT-2 recoveries were 90 %, 88 %, and 88 %; T-2 recoveries were 86 %, 89 %, and 86 %; and ZEA recoveries were 88 %, 90 %, and 87 %, respectively. The GC–MS method here reported for wheat beverage yielded recoveries of 96.10 % for DON, 97.18 % for HT-2, 103.38 % for T-2, and 72.49 % for ZEA. This consistency indicates that the GC–MS method provides quantitative performance comparable to established LC-MS/MS approaches. On the other hand, although LC-MS/MS methods often reach lower detection limits than GC–MS, the LODs reported here are sufficient to monitor mycotoxins in cereal-derived beverages. In addition, the present GC–MS method provides a short total runtime, high chromatographic resolution for structurally related trichothecenes, and relies on instrumentation that is widely accessible in routine testing laboratories. Taken together, these observations suggest that GC–MS and LC-MS/MS provide complementary analytical options, and that the validated GC–MS protocol presented here offers a reliable, selective, and cost-effective alternative for the simultaneous quantification of trichothecenes and zearalenone in wheat-based beverages.

## 4 Conclusion

A new GC–MS detection method for the rapid analysis of mycotoxins in wheat plant-based beverages has been developed. This method is based on a QuEChERS extraction procedure and able to identify 12 mycotoxins (FUS-X, NIV, DAS, HT-2, NEO, T-2 triol, T-2 tetraol, DON, 3-ADON, 15-ADON, T-2 and ZEA) in just 15 min. The method was validated in terms of linearity, LODs and LOQs, matrix effect, recoveries, and repeatability, exhibiting good linearity (R^2^ higher than 0.9992) and satisfactory recoveries range from 72.49 % to 114.57 %. The sample pre-treatment and the optimized QuEChERS minimized the matrix effect of such beverage reducing the interference of the presence of pigments and other compounds with the mycotoxins in the GC–MS analysis. The method is sensitive enough to detect 12 mycotoxins in wheat-based beverages in case these new matrices are included in the EU legislation. The method can detect both toxins (ZEA and DON) together with other 5 analogues (3-ADON, 15-ADON, T-2, DAS and HT-2) in the range of 62.5 μg/L-2 mg/L. Besides, other 5 mycotoxins are susceptible to be detected in similar ranges: 125 μg/L-3 mg/L for NIV, T-2 triol, and T-2 tetraol; 125 μg/L-4 mg/L for NEO; and 62.5 μg/L-3 mg/L for FUS-X, supporting its use as a screening multi-toxin method for mycotoxin detection in wheat beverages. The chemical structure of legislated mycotoxins can be modified by fungi, plant, or animal metabolism, leading to other non-legislated mycotoxins, like 3-ADON or 15-ADON which can be from DON. Data on these mycotoxins are still scarce, and therefore, it is necessary to develop multi-toxin methods for the detection of these compounds in novel matrices.

To summarize, in response to the mycotoxin contamination of wheat and its derivatives, which is of great concern in Europe, this study developed a new GC–MS method for the rapid extraction and determination of 11 TCTs and ZEA in wheat plant-based beverages.

## CRediT authorship contribution statement

**Ziyang Jia:** Writing – original draft, Methodology, Investigation, Data curation. **Wenqi Huang:** Investigation. **Kaifeng Zhao:** Methodology, Investigation. **Celia Costas:** Methodology, Investigation. **Maria Garcia-Marti:** Methodology, Investigation. **Jesus Simal-Gandara:** Resources, Funding acquisition. **Paz Otero:** Writing – review & editing, Validation, Supervision, Methodology, Investigation, Funding acquisition.

## Funding

The research leading to these results was supported WHEATBIOME granted by HORIZON-CL6–2022-FARM2FORK-01 (2023–2026) (grant agreement ID: 101084344), by MICINN for supporting the Ramón y Cajal's grant for Paz Otero (RYC2022–036690-I) and by Xunta de Galicia for supporting the program Excelencia-ED431F 2024/22 and GRC-ED431C 2022/25. Ziyang Jia is grateful for the financial support provided by the Program of China Scholarship Council (Grant No 202308420027), and Wenqi Huang and Kaifeng Zhao also acknowledges the support from the same Program (Grant No. 202206820002 and Gran No. 202408420059).

## Declaration of competing interest

The authors declare that they have no known competing financial interests or personal relationships that could have appeared to influence the work reported in this paper.

## Data Availability

Data will be made available on request.

## References

[bb0005] Arroyo-Manzanares N., Hamed A.M., García-Campaña A.M., Gámiz-Gracia L. (2019). Plant-based milks: Unexplored source of emerging mycotoxins. A proposal for the control of enniatins and beauvericin using UHPLC-MS/MS. Food Additives & Contaminants: Part B.

[bb0010] Bowden J.A., Colosi D.M., Mora-Montero D.C., Garrett T.J., Yost R.A. (2009). Enhancement of chemical derivatization of steroids by gas chromatography/mass spectrometry (GC/MS). Journal of Chromatography B.

[bb0015] Cunha S.C., Fernandes J.O. (2010). Development and validation of a method based on a QuEChERS procedure and heart-cutting GC-MS for determination of five mycotoxins in cereal products. Journal of Separation Science.

[bb0020] Desjardins A.E. (2009). From yellow rain to green wheat: 25 years of Trichothecene biosynthesis research. Journal of Agricultural and Food Chemistry.

[bb0025] Di Nunzio M., Pieri M., Gangitano D., Di Nunzio C., Tinto N., Niola M., Barrot-Feixat C. (2024). Leveraging genetics to support forensic toxicology analysis: Demonstrating concordance among marijuana samples. Journal of Applied Research on Medicinal and Aromatic Plants.

[bb0030] EC 2023/915 (2023). Commission Regulation (EU) 2023/915 of 25 April 2023 on maximum levels for certain contaminants in food and repealing Regulation (EC) No 1881/2006 (Text with EEA relevance) OJ L 119 05.05.2023, p. 103, ELI. http://data.europa.eu/eli/reg/2023/915/oj.

[bb0035] EC 2024/1022 (2024). Commission Regulation (EU) 2024/1022 of 8 April 2024 amending Regulation (EU) 2023/915 as regards maximum levels of deoxynivalenol in food OJ L, 2024/1022, 09.04.2024, ELI. http://data.europa.eu/eli/reg/2024/1022/oj.

[bb0040] EC 2024/1038 (2024). Commission Regulation (EU) 2024/1038 of 9 April 2024 amending Regulation (EU) 2023/915 as regards maximum levels of T-2 and HT-2 toxins in food OJ L, 2024/1038, 10.04.2024, ELI. http://data.europa.eu/eli/reg/2024/1038/oj.

[bb0045] Escrivá L., Font G., Manyes L. (2015). In vivo toxicity studies of fusarium mycotoxins in the last decade: A review. Food and Chemical Toxicology.

[bb0050] Ferreira I., Fernandes J.O., Cunha S.C. (2012). Optimization and validation of a method based in a QuEChERS procedure and gas chromatography–mass spectrometry for the determination of multi-mycotoxins in popcorn. Food Control.

[bb0055] Goswami R.S., Kistler H.C. (2004). Heading for disaster: Fusarium graminearum on cereal crops. Molecular Plant Pathology.

[bb0060] Hamed A.M., Abdel-Hamid M., Gámiz-Gracia L., García-Campaña A.M., Arroyo-Manzanares N. (2019). Determination of Aflatoxins in Plant-based Milk and Dairy Products by Dispersive Liquid–Liquid Microextraction and High-performance Liquid Chromatography with Fluorescence Detection. Analytical Letters.

[bb0065] Hamed A.M., Arroyo-Manzanares N., García-Campaña A.M., Gámiz-Gracia L. (2017). Determination of fusarium toxins in functional vegetable milks applying salting-out-assisted liquid-liquid extraction combined with ultra-high-performance liquid chromatography tandem mass spectrometry. Food Additives & Contaminants: Part A.

[bb0070] Hamed A.M., Arroyo-Manzanares N., García-Campaña A.M., Gámiz-Gracia L. (2017). Determination of fusarium toxins in functional vegetable milks applying salting-out-assisted liquid–liquid extraction combined with ultra-high-performance liquid chromatography tandem mass spectrometry. Food Additives & Contaminants: Part A.

[bb0075] Huang P., Yu X., Liu H., Ding M., Wang Z., Xu J.-R., Jiang C. (2024). Regulation of TRI5 expression and deoxynivalenol biosynthesis by a long non-coding RNA in fusarium graminearum. Nature Communications.

[bb0080] Juan C., Mañes J., Font G., Juan-García A. (2017). Determination of mycotoxins in fruit berry by-products using QuEChERS extraction method. LWT.

[bb0085] Kerstner F., Garda-Buffon J. (2024). Mycotoxins in plant-based beverages: An updated occurrence. Food Research International.

[bb0090] Lawrence S.E., Lopetcharat K., Drake M.A. (2016). Preference mapping of soymilk with different U.S. Consumers. Journal of Food Science.

[bb0095] Liu H., Luo J., Kong W., Liu Q., Hu Y., Yang M. (2016). UFLC-ESI-MS/MS analysis of multiple mycotoxins in medicinal and edible Areca catechu. Chemosphere.

[bb0100] Mahmoud A.F., Escrivá L., Rodríguez-Carrasco Y., Moltó J.C., Berrada H. (2018). Determination of trichothecenes in chicken liver using gas chromatography coupled with triple-quadrupole mass spectrometry. LWT.

[bb0105] Miró-Abella E., Herrero P., Canela N., Arola L., Borrull F., Ras R., Fontanals N. (2017). Determination of mycotoxins in plant-based beverages using QuEChERS and liquid chromatography–tandem mass spectrometry. Food Chemistry.

[bb0110] Penha C.B., Santos V.D.P., Speranza P., Kurozawa L.E. (2021). Plant-based beverages: Ecofriendly technologies in the production process. Innovative Food Science & Emerging Technologies.

[bb0115] Proctor R.H., McCormick S.P., Gutiérrez S. (2020). Genetic bases for variation in structure and biological activity of trichothecene toxins produced by diverse fungi. Applied Microbiology and Biotechnology.

[bb0120] Rai A., Mukul D., Tripathi A. (2020). Occurrence and toxicity of a fusarium mycotoxin, zearalenone. Critical Reviews in Food Science and Nutrition.

[bb0125] Ran R., Wang C., Han Z., Wu A., Zhang D., Shi J. (2013). Determination of deoxynivalenol (DON) and its derivatives: Current status of analytical methods. Food Control.

[bb0130] Rodríguez-Carrasco Y., Berrada H., Font G., Mañes J. (2012). Multi-mycotoxin analysis in wheat semolina using an acetonitrile-based extraction procedure and gas chromatography–tandem mass spectrometry. Journal of Chromatography A.

[bb0135] Rodríguez-Carrasco Y., Font G., Mañes J., Berrada H. (2013). Determination of Mycotoxins in Bee Pollen by Gas Chromatography–Tandem Mass Spectrometry. Journal of Agricultural and Food Chemistry.

[bb0140] Rodríguez-Carrasco Y., Moltó J.C., Berrada H., Mañes J. (2014). A survey of trichothecenes, zearalenone and patulin in milled grain-based products using GC–MS/MS. Food Chemistry.

[bb0145] Stahr H.M., Domoto M., Hyde W., Martin P.J. (1985). Volatilization of trichothecene mycotoxins for analysis. Microchemical Journal.

[bb0150] Suzuki T., Iwahashi Y. (2012). Comprehensive gene expression analysis of type B Trichothecenes. Journal of Agricultural and Food Chemistry.

[bb0155] Willms T., Kryk H., Hampel U. (2016). The gas chromatographic analysis of the reaction products of the partial isobutane oxidation as a two phase process. Journal of Chromatography A.

[bb0160] Yang X., Liu P., Cui Y., Xiao B., Liu M., Song M., Li Y. (2020). Review of the reproductive toxicity of T-2 toxin. Journal of Agricultural and Food Chemistry.

[bb0165] Zhang D., Bond T., Pan Y., Li M., Luo J., Xiao R., Chu W. (2022). Identification, occurrence, and cytotoxicity of Haloanilines: A new class of aromatic nitrogenous disinfection byproducts in Chloraminated and chlorinated drinking water. Environmental Science & Technology.

[bb0170] Zhou J., Zhao L., Huang S., Liu Q., Ao X., Lei Y., Ji C., Ma Q. (2022). Zearalenone toxicosis on reproduction as estrogen receptor selective modulator and alleviation of zearalenone biodegradative agent in pregnant sows. Journal of Animal Science and Biotechnology.

